# Genetic characterization and functional analysis of novel PITX2 variants identified in Chinese families with Axenfeld-Rieger syndrome: PITX2 variants in Chinese ARS and mechanisms

**DOI:** 10.3724/abbs.2025167

**Published:** 2025-10-09

**Authors:** Junqin Xu, Xinyao Wang, Zilin Zhong, Jianjun Chen, Peng Yang

**Affiliations:** 1 Shanghai Key Laboratory of Anesthesiology and Brain Functional Modulation Clinical Research Center for Anesthesiology and Perioperative Medicine Translational Research Institute of Brain and Brain-Like Intelligence Shanghai Fourth People’s Hospital School of Life Sciences and Technology Tongji University Shanghai 200092 China; 2 Shanghai Key Laboratory of Anesthesiology and Brain Functional Modulation Clinical Research Center for Anesthesiology and Perioperative Medicine Translational Research Institute of Brain and Brain-Like Intelligence Department of Pediatrics Shanghai Fourth People’s Hospital School of Medicine Tongji University Shanghai 200434 China; 3 Institute of Medical Genetics Department of Big Data in Health Science School of Public Health and General Practice Medicine School of Medicine Tongji University Shanghai 200092 China; 4 Tongji University School of Medicine Shanghai 200331 China

Axenfeld-Rieger syndrome (ARS), initially characterized by Theodor Axenfeld and Herwigh Rieger in the early 20th century [1], is a rare genetic disorder with an estimated prevalence of 1:200,000 in live births [2]. This condition is characterized by distinctive anterior segment dysgenesis, including iris stromal hypoplasia, corectopia, and posterior embryotoxon. It is also accompanied by characteristic systemic features such as craniofacial dysmorphism, dental anomalies, cardiovascular malformations, and periumbilical skin redundancy [ 3, 4].

Genetically, ARS is highly heterogeneous, with pathogenic variants identified in several genes. Among these, *PITX2* on chromosome 4q25 [5] and *FOXC1* on chromosome 6p25 [6] are the most frequently implicated. In particular, *PITX2* is a transcription factor harboring a highly conserved homeobox domain, which regulates the transcription of downstream targets essential for ocular, dental, cardiac, and umbilical development [ 7, 8]. Variants in *PITX2* may alter protein localization or impair its transcriptional activity, thereby disrupting normal developmental processes and leading to the characteristic phenotypes of ARS [ 9, 10].

Despite its clinical and genetic significance, ARS is thought to be underdiagnosed, and most studies on *PITX2*-related ARSs remain limited to individual case reports. In the present study, we aimed to delineate the genetic spectrum of ARS in Chinese patients and to elucidate the molecular mechanisms underlying the pathogenesis associated with *PITX2* variants.

Four unrelated Chinese families affected by the ARS were recruited for this study. This study was approved by the Institutional Review Board of Tongji University School of Life Sciences and Technology (Shanghai, China) and was conducted in accordance with the ethical principles of the Declaration of Helsinki. Approximately 5 mL of peripheral venous blood was collected from the probands and their family members using EDTA-containing vacutainer tubes. Genomic DNA was extracted using a blood DNA extraction kit (Tiangen, Beijing, China) following the manufacturer’s protocol. Specific primers for *PITX2* were subsequently designed ( Supplementary Table S1), and the PCR products from the blood samples of the four family members were analyzed by Sanger sequencing.

Four heterozygous *PITX2* variants were identified in Chinese ARS-affected families, all of which co-segregated with characteristic ocular phenotypes. The first proband was a 20-year-old female carrying a frameshift mutation in the *PITX2* gene (c.118delA, p.Arg40Glyfs*115). Clinically, the patient presented with corneal opacity and glaucoma, accompanied by systemic developmental anomalies, including sparse and irregular teeth and an umbilical protrusion ( Figure 1A,B). The proband’s mother (II:1) was a heterozygous carrier of this mutation. However, the proband’s parents (II:4, II:5) and maternal grandmother (I:2) did not carry the mutation ( Figure 1B). The second proband carried a nonsense mutation in the *PITX2* gene (c.211G > T, p.Glu71X). Genetic sequencing of the family members revealed that the proband’s spouse was not a carrier of this mutation, while the mother was a heterozygous carrier, and the father did not carry the mutation ( Figure 1C). By analyzing the available family members with *PITX2* sequences, we determined that the inheritance pattern follows an autosomal dominant mode, affecting both males and females.

**Figure 1:**
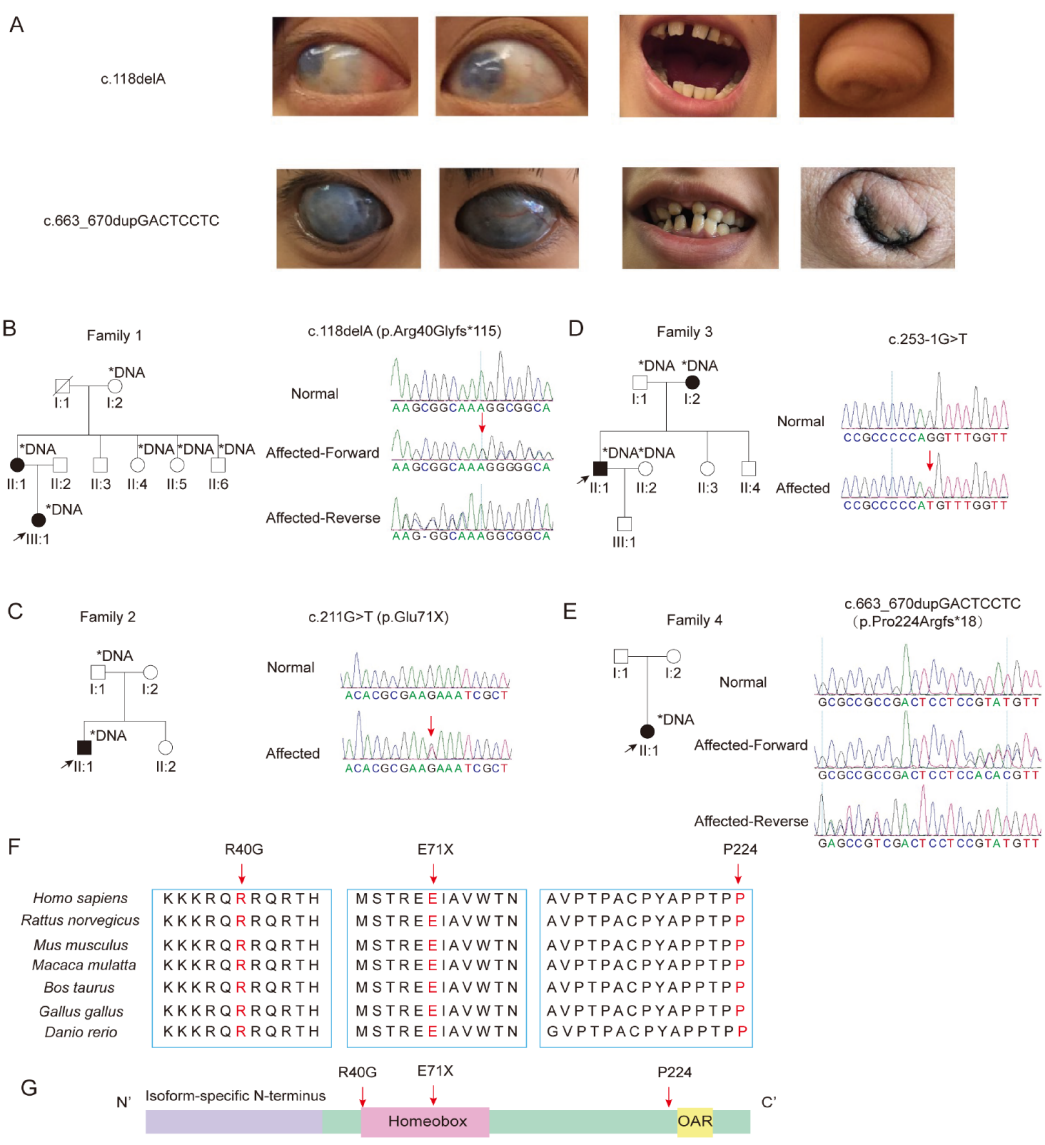
Figure 1 Clinical symptoms of ARSs and pedigrees of the four families (A) Clinical manifestations of ARS. (B–E) Pedigrees of the four ARS probands and their Sanger sequencing results. ↗: Proband; ■: affected male; ●: affected female; □: unaffected male; ○: unaffected female. (F) Conservation analysis of PITX2 mutation sites. (G) Schematic diagram of the PITX2 functional domains and mutation sites.

The third proband (II:1) exhibited a specific deletion and point mutation in *PITX2* (c.253-1G > T), and sequencing revealed that the father did not carry this mutation ( Figure 1D). The fourth proband carried a frameshift mutation in the *PITX2* gene (c.663_670dupGACTCCTC, p.Pro224Argfs*18). Clinically, this patient exhibited more severe corneal opacity and glaucoma, as well as sparse teeth and a darker umbilical color ( Figure 1A). Both parents showed no apparent clinical phenotype ( Figure 1E). These results indicate that the variants were not present in the parents’ genomes, suggesting that these two variants arose *de novo* during development. Therefore, the disease also has sporadic characteristics.

These variants were not found in the ExAC and gnomAD databases, indicating that they are likely pathogenic variants rather than genetic polymorphisms. In addition to the previously reported *PITX2* variant (c.211G > T, p.Glu71X) [11], the other three variants are novel in the Chinese population ( Supplementary Table S2).

Evolutionary conservation analysis revealed that these variants occur at highly conserved amino acid residues, suggesting their critical biological roles and potential pathogenicity ( Figure 1F). The R40G and E71X variants are located within or near the homeobox domain, which is essential for DNA binding, whereas the P224 variant is situated close to the OAR domain, which participates in transcriptional regulation. The localization of these variants within functionally relevant regions further suggests that these variants are likely to disrupt PITX2 protein function ( Figure 1G and Supplementary Figure S1A). Analysis of the Leiden Open Variation Database 3.0 (LOVD3) database and published literature indicates that 40% of reported variants occur in the homeobox domain and that 7% occur in the OAR domain ( Supplementary Figure S1B). Splicing errors predominantly cluster within the intron between exon 5 and exon 6 ( Supplementary Figure S1C), a region critical for proper expression of the homeobox domain. Notably, the newly identified *PITX2* variant (c.253-1G > T) is also located within this key region ( Supplementary Figure S1C), highlighting its potential impact on protein function.

Genomic DNA sequencing revealed that the *PITX2* variant (c.253-1G > T) resulted in the loss of the splice acceptor site ( Supplementary Figure S2A). To validate how this variant affects the splicing of *PITX2*, wild-type (WT) and mutant PITX2 constructs were cloned and inserted into the pCAS2 plasmid [12] and transfected into cells. RT-PCR analysis revealed that the mutant PITX2 generated a smaller splicing product ( Figure 2A). Sanger sequencing confirmed that the variant produced at least two abnormal splice isoforms: one with a 431 bp deletion (c.del253–683), leading to a premature termination codon (PTC), and another with a 12 bp deletion (c.del253–264), resulting in an in-frame deletion of four amino acids within the VWFK motif ( Figure 2B).

**Figure 2:**
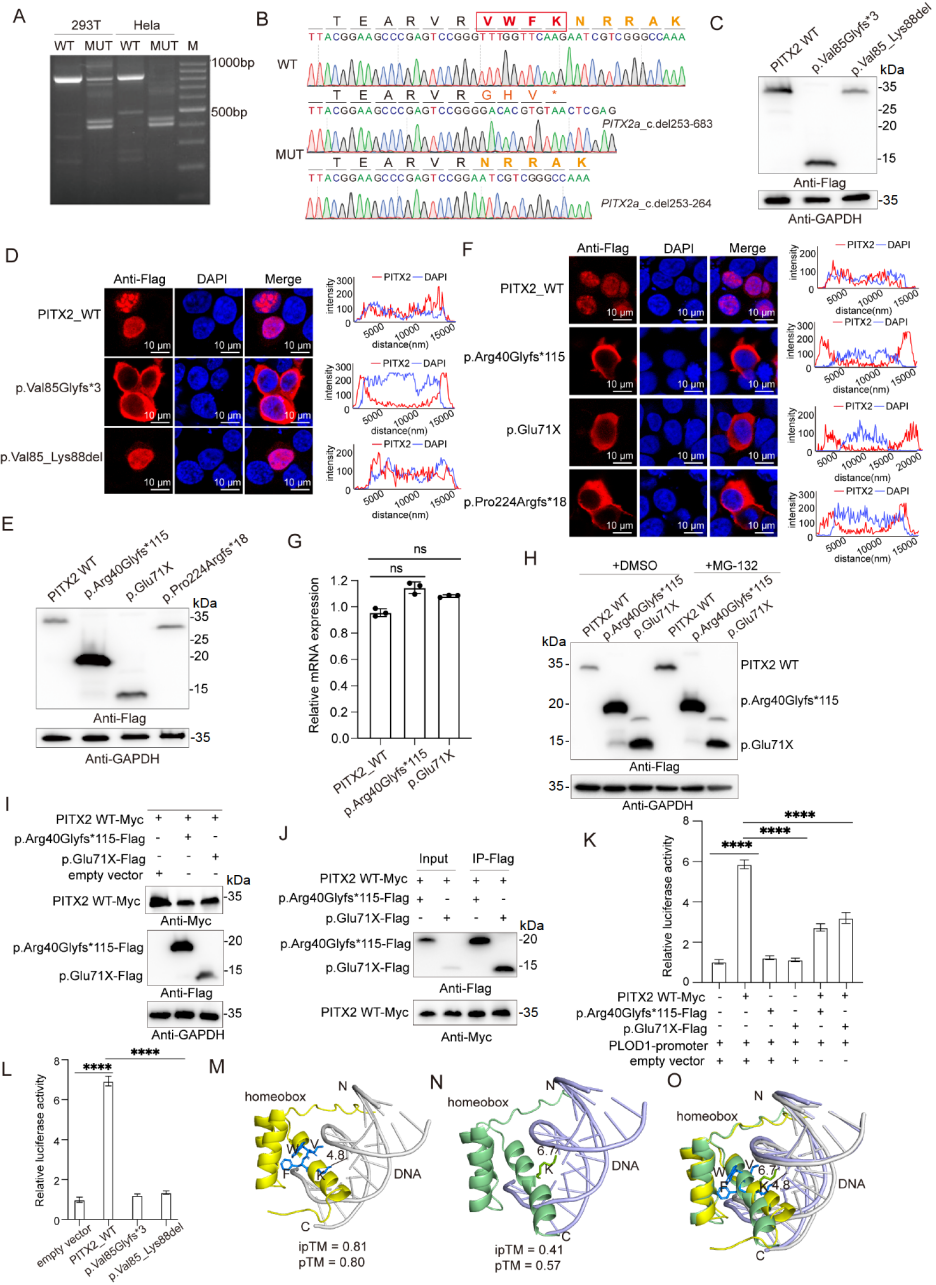
Figure 2 Functional characterization and structural impact of the *PITX2* variants (A) RT-PCR analysis of splicing products in HEK293T and HeLa cells transfected with WT or mutant PITX2 plasmids. (B) Sanger sequencing was used to validate the sequence of the splicing products. (C,E) Western blot analysis of mutant PITX2 protein expression levels. (D,F) Subcellular localization of the WT and mutant PITX2 proteins. Flag-tagged proteins were visualized by immunofluorescence and confocal microscopy (RFP signal), with the nuclei counterstained with DAPI (blue). Scale bar: 10 μm. The fluorescence intensity profiles of the PITX2 and DAPI signals are shown on the right. (G) qPCR analysis of the mRNA expression levels of PITX2 variants. Student’s t test was performed for statistical analysis. The error bars represent the standard deviation (SD). *P < 0.05, **P < 0.01, ***P < 0.001, ****P < 0.0001, and P > 0.05 (n.s). Sample size: n = 3. (H) Western blot analysis of protein levels following the treatment of HEK293T cells with 10 μM MG-132 for 6 h. (I) Western blot analysis of PITX2 WT protein expression after co-transfection with mutant constructs. (J) Co-immunoprecipitation was performed via the use of Flag beads to assess interactions between WT and mutant proteins. (K,L) Dual-luciferase reporter assay was used to evaluate the effect of variants on PITX2 WT transcriptional activity. Student’s t test was performed for statistical analysis. The error bars represent the standard deviation (SD). *P < 0.05, ** P < 0.01, ***P < 0.001, ****P < 0.0001, and P > 0.05 (ns). Sample size: n = 3. (M) Predicted structure of the PITX2 WT homeodomain (yellow) bound to the TAATCC DNA sequence (gray), with VWFK residues highlighted in blue. (N) Predicted structure of the mutant homeodomain lacking VWFK residues (green) bound to DNA (light blue). (O) Structural alignment of WT and VWFK-deleted PITX2 homeodomains bound to the TAATCC DNA sequence.

To investigate these splice variants at the protein level, p.Val85Glyfs*3 and p.Val85_Lys88del constructs were transiently transfected into HEK293T cells. Western blot analysis revealed that p.Val85Glyfs*3 was expressed at levels comparable to those of WT PITX2, whereas p.Val85_Lys88del presented reduced protein levels ( Figure 2C and Supplementary Figure S2B). Immunofluorescence analysis revealed the nuclear-to-cytoplasmic mis-localization of p.Val85Glyfs*3, whereas p.Val85_Lys88del retained nuclear localization ( Figure 2D and Supplementary Figure S2C).

To examine the effects of the truncating variants, the p.Arg40Glyfs*115, p.Glu71X, and p.Pro224Argfs*18 constructs were transiently expressed in HEK293T cells. Western blot analysis revealed that all three variants produced truncated proteins. Notably, p.Arg40Glyfs*115 and p.Glu71X presented higher expression levels than did PITX2 WT, with p.Arg40Glyfs*115 showing the most pronounced increase, whereas p.Pro224Argfs*18 presented reduced protein expression ( Figure 2E and Supplementary Figure S2D). Immunofluorescence analysis demonstrated the nuclear-to-cytoplasmic mis-localization for of p.Arg40Glyfs*115 and p.Glu71X; p.Pro224Argfs*18 also displayed cytoplasmic retention ( Figure 2F and Supplementary Figure S2E).

To investigate the mechanism underlying the increased protein levels of p.Arg40Glyfs*115 and p.Glu71X, qPCR analysis revealed that their mRNA levels were comparable to those of *PITX2* WT, indicating that the observed increase in protein levels was not attributable to transcriptional differences ( Figure 2G). Treatment with the proteasome inhibitor MG-132 led to the accumulation of WT PITX2 but had no effect on the mutants, suggesting that the mutants presented enhanced stability relative to that of WT PITX2 ( Figure 2H and Supplementary Figure S2F). Co-expression of PITX2 WT and mutant constructs at equal plasmid amounts resulted in reduced PITX2 WT protein levels, implying a potential dominant-negative effect ( Figure 2I and Supplementary Figure S2G). Co-immunoprecipitation (co-IP) revealed interactions between the mutants and the WT PITX2 protein ( Figure 2J and Supplementary Figure S2H). Dual-luciferase reporter assays demonstrated that PITX2 increased transactivation of the *PLOD1* promoter sixfold, whereas p.Arg40Glyfs*115 and p.Glu71X showed no transactivation activity. Moreover, co-expression of these variants reduced the transcriptional activation of PLOD1 by WT PITX2 ( Figure 2K). Collectively, these results indicate that the p.Arg40Glyfs*115 and p.Glu71X variants exhibit increased protein stability and exert a partial dominant-negative effect by interacting with WT PITX2, leading to reduced WT-mediated transcriptional activation.

Structural modelling revealed that helix 3 of the wild-type PITX2 homeodomain closely contacts the major groove of DNA, with Lys88 (K50 of the homeobox) positioned 4.8 Å from the fourth thymine of the TAATCC motif ( Figure 2M). Deletion of the VWFK motif (p.Val85_Lys88del) induced a C-terminal shift of helix 3, increasing the distance between Lys93 and DNA to 6.7 Å, which likely weakens DNA binding and reduces the confidence of the predicted complex ( Figure 2N,O). Dual-luciferase assays revealed that deletion of the VWFK motif abolished PITX2 transactivation activity ( Figure 2L), indicating that this region is critical for DNA binding. NMR analysis by Chaney *et al*. [13] indicated that K50 and its interaction with DNA are highly sensitive to the spatial positioning of helix 3 and that conformational displacement likely disrupts the hydrogen-bonding network between K50 and DNA. Notably, the splice-altered protein and the two stability-enhanced variants lack this critical VWFK region ( Supplementary Figure S3), suggesting a compromised DNA-binding capacity.

In conclusion, the four PITX2 variants identified in this study are pathogenic through distinct mechanisms. The c.253-1G > T variant disrupts normal splicing, producing proteins with mis-localization or reduced dosage. p.Pro224Argfs*18 is expressed at low levels and has cytoplasmic retention. Both p.Arg40Glyfs*115 and p.Glu71X are stabilized but exert dominant-negative effects that inhibit WT-mediated transcription. Deletion of the VWFK motif impairs DNA binding and transactivation activity. These findings expand the genetic spectrum of ARS and provide mechanistic insights that may inform precise clinical diagnosis and the development of targeted therapies.

## Supporting information

25604supplementary-z(1)

## Supplementary Data

Supplementary data are available at *Acta Biochimica et Biophysica Sinica* online.
