# Supplementary material for: Genetic characterization and functional analysis of novel PITX2 variants identified in Chinese families with Axenfeld-Rieger syndrome: PITX2 variants in Chinese ARS and mechanisms
Source: Acta Biochim Biophys Sin (Shanghai). 2025 Oct 9;58(5):1179–82. doi: 10.3724/abbs.2025167 (PMC13191470; doi:10.3724/abbs.2025167)
Supplement: 25604supplementary-z(1) [file 25604supplementary-z(1).docx]

**Supplementary Materials and Methods**

**Minigene assay**

To investigate the potential splicing effects of the variants, an *in vitro* minigene splicing assay was conducted. The splicing plasmid of the pCAS2 vector was a gift from Professor Alexandra Martins [1]. The exons and 150 bp of the introns of *PITX2* were inserted into the vector. For the c.253-1G>T variant, the plasmid was obtained using a Recombinant Cloning kit (LABLEAD, Beijing, China). Thirty-six hours post-transfection with the wild-type or mutant plasmid, the HEK293T and HeLa cells were collected. Total RNA was extracted using Trizol (Tiangen, Beijing, China). Approximately 1 μg of total RNA (OD260/280 = 2.0) was reverse-transcribed into first-strand cDNA using the FastKing one-step RT-PCR kit (Tiangen) and stored at −20°C until further use. The specific primers used for amplification are detailed in **Supplementary Table S1**. Subsequent sequencing was performed to determine the changes in gene sequences resulting from mRNA splicing.

**Plasmid construction**

The coding region of PITX2 was amplified from cDNA by PCR using a high-fidelity DNA polymerase, generating both wild-type and mutant coding sequences. The pCDNA3.1^+^ vector was linearized by *Xho*I and *Bam*HI digestion at 37°C for 1 h. The amplified PITX2 fragments were then assembled into the linearized vector using DNA Assembly Mix Plus (LABLEAD) to generate wild-type and variant plasmids with different tags. Both the wild-type and variant plasmids were subjected to sequencing to confirm the inserted sequences and target mutations.

**Cell culture and transfection**

HeLa and HEK293T cells were cultured in high-glucose Dulbecco’s modified Eagle medium (DMEM; Gibco, Carlsbad, USA) supplemented with 10% fetal bovine serum (FBS; Gibco) and incubated at 37°C in a humidified atmosphere with 5% CO_2_. The cells were seeded one day prior to transfection, and transfection was performed the next day when the cell density reached 50%−60%. The transfection was performed using Vigofect Transfection Reagent (Vigorous, Shanghai, China) according to the manufacturer’s protocol.

**Co-immunoprecipitation (co-IP)**

For co-IP, HEK293T cells were co-transfected with Flag-tagged PITX2 mutant or Myc-tagged PITX2 wild-type (WT) plasmids. After 36 h, the cells were pelleted and washed twice with cold PBS, and the proteins were extracted in RIPA buffer or IP buffer [20 mM Tris, pH 7.5, 150 mM NaCl, 1% Triton X-100, and proteinase inhibitor cocktail (PIC)] (Roche, Basel, Switzerland). The cell extracts were incubated with protein A/G magnetic beads (Thermo Fisher Scientific, Waltham, USA) crosslinked with anti-FLAG antibodies (F1804; Sigma-Aldrich, St Louis, USA) at 4°C overnight. The beads were then washed three times with BC150 buffer (10 mM Tris pH 7.8, 0.5 mM EDTA, 10% glycerol, 150 mM NaCl, and protease inhibitor cocktail), and the bound proteins were eluted by boiling in 1× loading buffer.

**Western blot analysis**

For western blot analysis, 36 h post-transfection of HEK293T cells with WT or mutant plasmids, the cells were lysed on ice using RIPA lysis buffer (Beyotime Biotech, Shanghai, China) supplemented with protease inhibitors. Total proteins were extracted from the supernatant following centrifugation at 12,000 rpm for 15 min at 4°C. Protein samples were denatured with SDS-PAGE loading buffer (Epizyme Biotech, Shanghai, China), separated by 15% SDS-PAGE, and transferred onto polyvinylidene fluoride (PVDF) membranes. The membranes were blocked with 5% non-fat milk for 1 h at room temperature, washed three times with TBST, and incubated overnight at 4°C with anti-Flag (1:1000 dilution; Sigma-Aldrich) or anti-Myc (1:1000 dilution; Sangon, Shanghai, China) and anti-GAPDH monoclonal antibodies (1:5000 dilution; Sangon Biotech) to detect PITX2-Flag recombinant proteins. The membranes were subsequently incubated with a secondary antibody (goat anti-mouse IgG; 1:5000 dilution; Invitrogen, Waltham, USA) for 1 h. Finally, the protein bands were visualized using a chemiluminescent western blot substrate (Thermo Fisher Scientific) on a Bio-Rad imaging system (Bio-Rad Laboratories, Hercules, USA). The fluorescence intensities of the western blot bands were quantified using Image Lab, and the normalized values were plotted as bar graphs via GraphPad Prism.

**Immunofluorescence staining**

HEK293T cells were seeded onto coverslips in 24-well plates and cultured in DMEM supplemented with 10% FBS. Twenty-four hours post-transfection, the culture medium was carefully aspirated. The cells were washed three times with phosphate-buffered saline (PBS) to remove phenol red and residual medium. The cells were fixed by the addition of 4% paraformaldehyde (PFA) and incubated at 4°C overnight. After fixation, the cells were washed three times with PBS to remove residual PFA. The cells were then permeabilized with 0.5% Triton X-100 for 15 min, followed by blocking with 3% BSA containing 0.15% Triton X-100 at room temperature for 2 h. The cells were then incubated overnight at 4°C with a 1:100 dilution of anti-Flag primary antibody (1:1000 dilution; Sigma-Aldrich), followed by incubation with a 1:500 dilution of Alexa Fluor® Plus 594 (Thermo Fisher Scientific) secondary antibody at room temperature for 2 h. After washing three times with TBST, the cell nuclei were stained with DAPI dye (0.5 μg/mL) for 1 min. The cells were washed three times with TBST to remove excess DAPI. The subcellular localization of WT and mutant PITX2 proteins was visualized using a confocal fluorescence microscope at 63× magnification. In the ZEN software of ZEISS confocal microscopy, the profile analysis function was used to extract fluorescence intensity distribution data along linear regions of interest in images.

**qPCR quantitation**

Total RNA was extracted 36 h post-infection using the phenol–chloroform method. Approximately 1 µg of total RNA was reverse-transcribed into first-strand cDNA via the FastKing One-Step RT-PCR Kit (Tiangen). The resulting cDNA was diluted 1:10 with nuclease-free water and used as the template for quantitative PCR (qPCR) with MagicSYBR Mixture (CWBio, Beijing, China). The primer sequences are provided in **Supplementary Table S1**. The qPCRs were performed at 95°C for 2 min, followed by 20 cycles of 95°C for 15 s, 60°C for 10 s, and 72°C for 3 min.

**Luciferase assays**

For the luciferase assays, annealed oligos (5′- gatccatcTAATCCcgtcgTAATCCgatggatc-3′) and the *PLOD1* promoter were cloned and inserted into the pGL3-SV40 vector. These pGL3 reporter plasmids were cotransfected with different PITX2 vectors (cloned and inserted into the pcDNA3.1 vector) into HEK293T cells using Vigofect (Vigorous) according to the manufacturer’s protocol together with the *Renilla* luciferase-expressing pRL-SV40 vector (#27163; Addgene, Cambridge, USA) for internal normalization. The cells were seeded in 96-well plates and transfected with 180 ng of PITX2 plasmid, 60 ng of pGL3-SV40 reporter, and 10 ng of pRL-TK. For the dominant-negative assay, the transfection mixture contained 100 ng of PITX2 WT plasmid, 100 ng of PITX2 mutant plasmid, 40 ng of pGL3-SV40 reporter plasmid, and 10 ng of pRL-TK control plasmid. Luciferase activity was measured 36 h after transfection using this dual-luciferase reporter assay system (Promega, Madison, USA).

**Prediction of DNA-protein interactions via AlphaFold3**

Structural models of the PITX2 homeodomain and its variants in complex with the DNA motif 5′-TAATCC-3′ were predicted via the AlphaFold server [2] under default parameters. Protein sequences (UniProt ID: Q99697) and the DNA motif were supplied as FASTA inputs. The resulting protein-DNA complexes were visualized and analyzed using PyMOL.


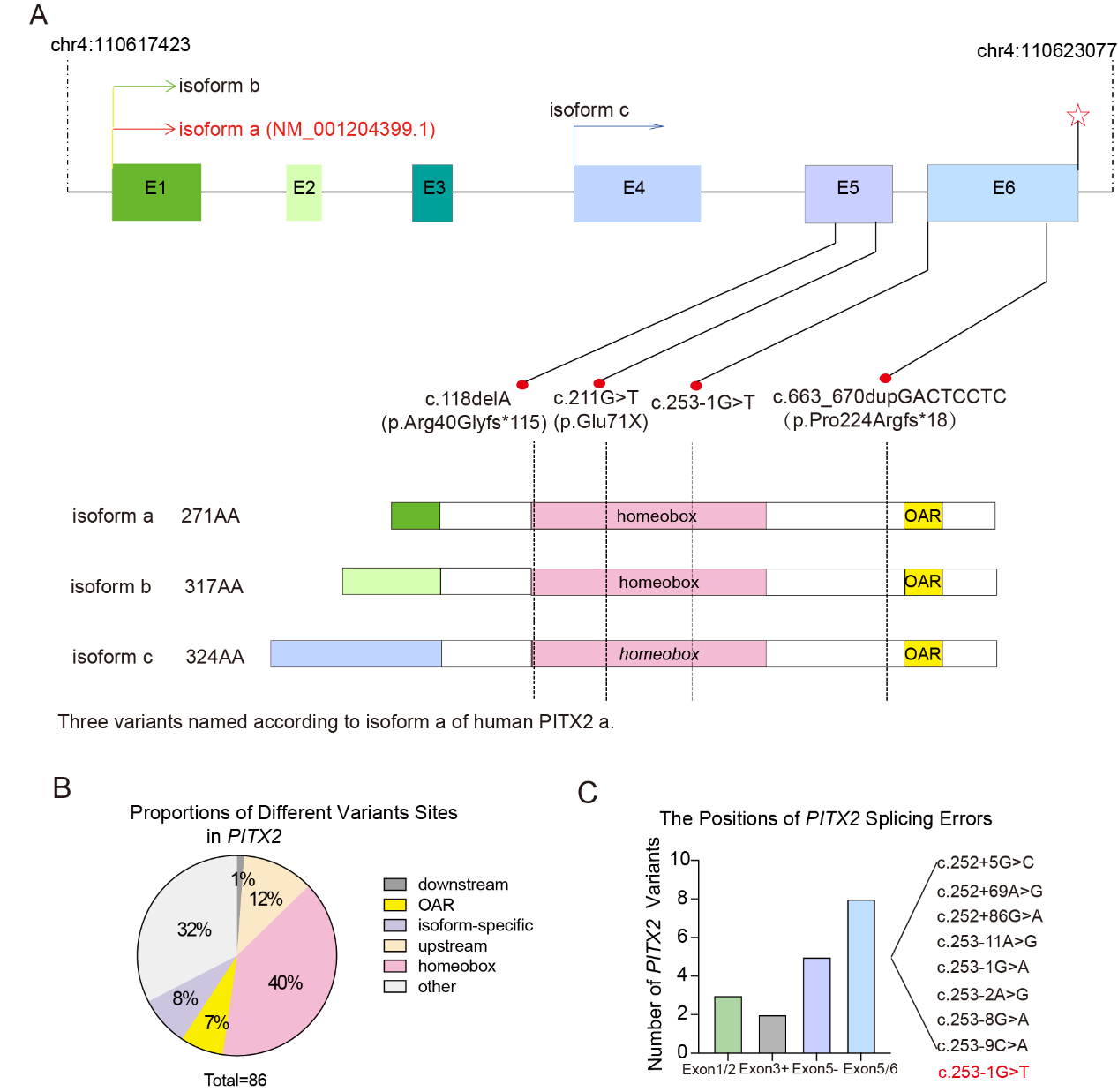


**Supplementary Figure S1**. **Genomic distribution and frequency of *PITX2* variants** (A) Distribution of variants across PITX2 isoforms. (B) Pie chart showing the proportions of PITX2 variants in different functional regions. (C) Bar plot showing the frequency of PITX2 splicing errors at different genomic loci; the newly identified variant (red) was excluded from the statistical analysis. Exon1/2: variants located in introns flanking Exon 1 or Exon 2; Exon3+: variants in the intron downstream of Exon 3; Exon5−: variants in the intron upstream of Exon 5; and Exons5/6: variants in the intron between Exon 5 and Exon 6.

**
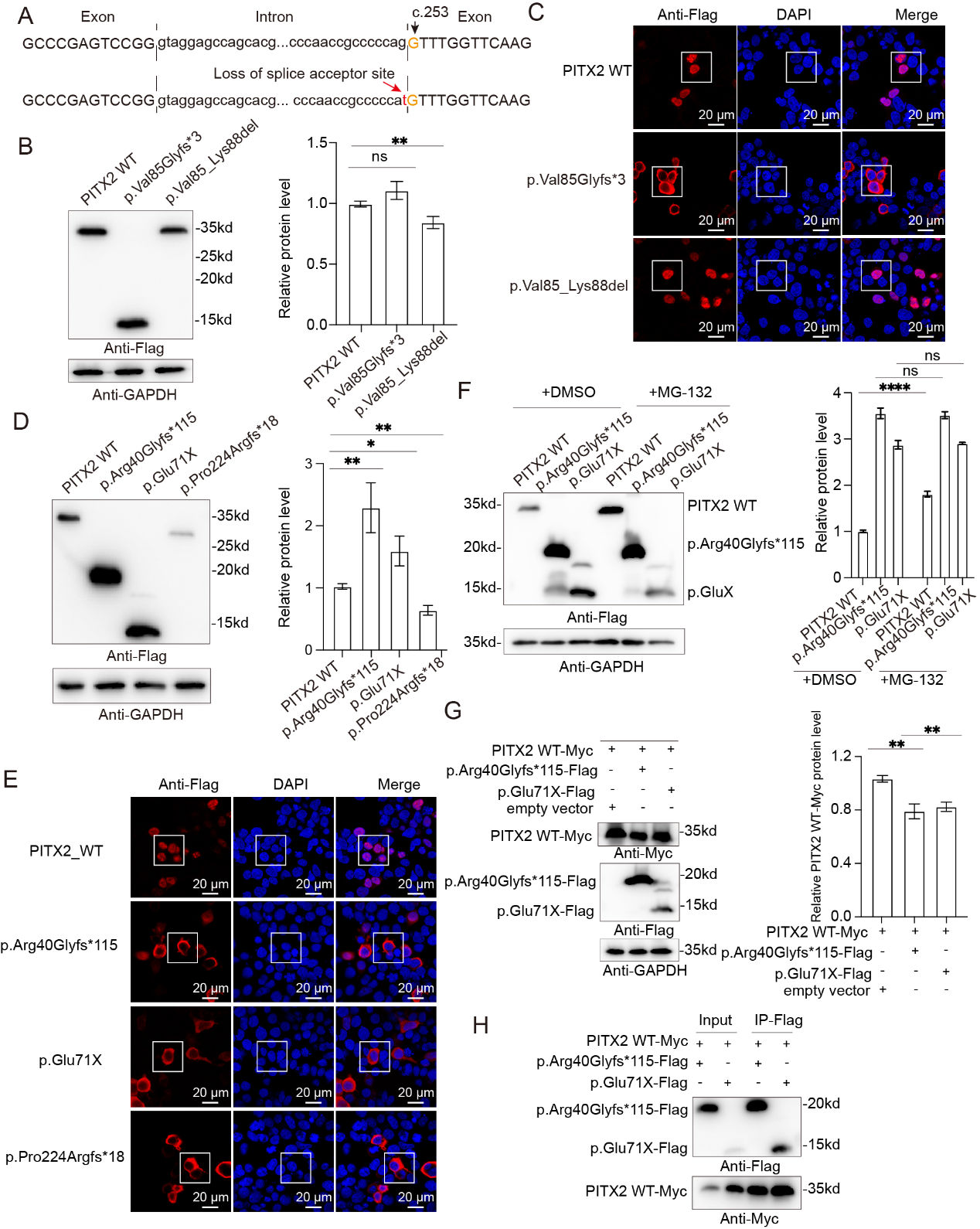
**

**Supplementary Figure S2. Functional characterization of *PITX2* variants** (A) The *PITX2* variant (c.253-1G>T) leads to aberrant RNA splicing. (B,D) Western blot analysis of mutant PITX2 protein expression levels. These panels represent the biological replicates shown in (C,E). The quantification of protein levels is shown in the bar graphs on the right. Statistical analysis was performed using Student’s *t* test; the error bars represent the standard deviation (SD), **P* < 0.05, ***P* < 0.01, ****P* < 0.001, *****P* < 0.0001, and *P* > 0.05 (n.s). Sample size: *n* = 3. (C,E) Subcellular localization of the WT and mutant PITX2 proteins. Flag-tagged proteins were detected by immunofluorescence staining and confocal microscopy (RFP signal), with the nuclei counterstained with DAPI (blue). Scale bar, 20 μm. Boxed regions correspond to the enlarged views shown in (D,F). (F) Western blot analysis of protein levels after treatment of HEK293T cells with 10 μM MG-132 for 6 h, representing a biological replicate of the data in (H). The quantification of protein levels is shown in the bar graphs on the right. Statistical analysis was performed via Student’s *t* test; the error bars represent SD, **P* < 0.05, ***P* < 0.01, ****P* < 0.001, *****P* < 0.0001, and *P* > 0.05 (n.s). Sample size, *n* = 3. (G) Western blot analysis of PITX2 WT protein expression after co-transfection with mutant constructs, representing a biological replicate of the data in (I). The quantification of protein levels is shown in the bar graphs on the right. Statistical analysis was performed using Student’s *t* test; the error bars represent SD, **P* < 0.05, ***P* < 0.01, ****P* < 0.001, *****P* < 0.0001, and *P* > 0.05 (n.s). Sample size, *n* = 3. (H) Co-immunoprecipitation using Flag beads to assess interactions between WT and mutant proteins, representing a biological replicate of the data in (J).


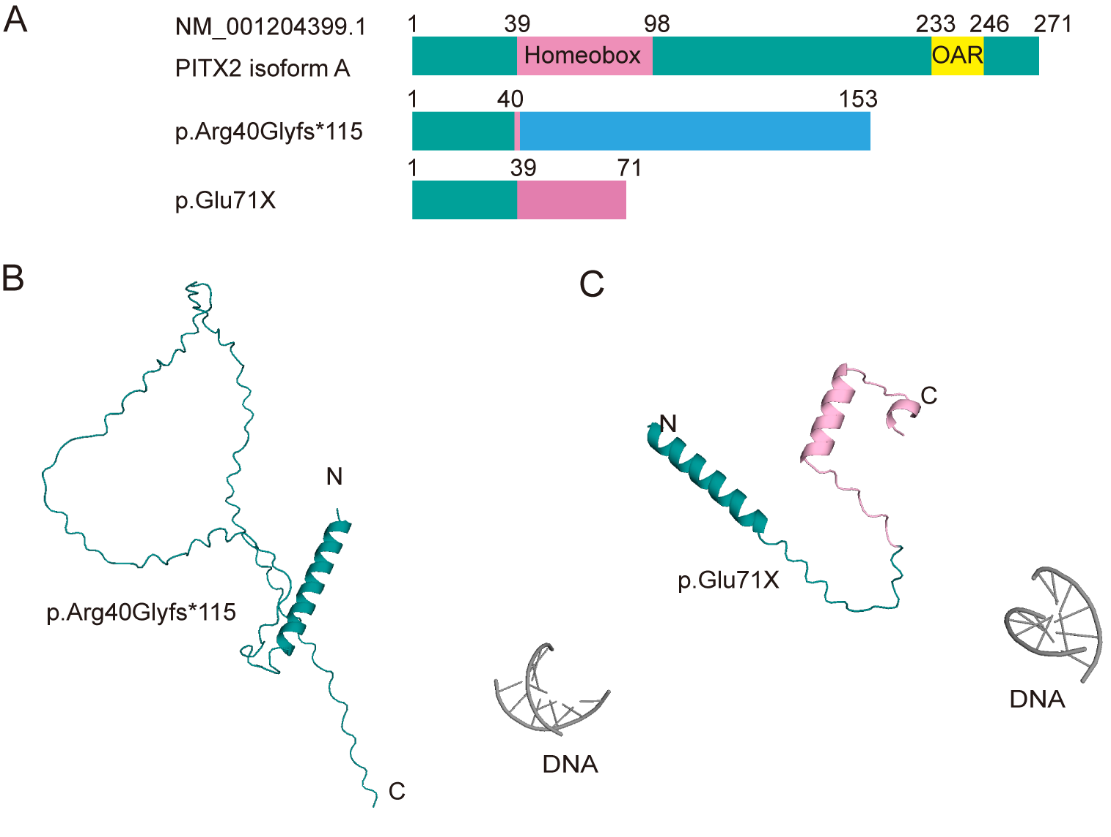


**Supplementary Figure S3. Structural analysis of p.Arg40Glyfs*115 and p.Glu71X** (A) Schematic diagrams of different PITX2 mutant proteins. (B,C) Predicted structures of p.Arg40Glyfs*115 (B) and p.Glu71X (C) in complex with the DNA motif TAATCC, illustrating the loss of the DNA-binding homeodomain and potential impact on DNA interaction.

**Supplementary Table S**1. **The sequences of primers used in the study**

| **Primers for screening *PITX2* variants** | |
| --- | --- |
| PITX2-EX2F | 5′-CCTGAAGCCTAGCACACAGTA-3′ |
| PITX2-EX2R | 5′-GAGGGACAAAGAGCAAAGACC-3′ |
| PITX2-EX3F | 5′-CAGAAAGAGTACGCCATCCTG-3′ |
| PITX2-EX3R | 5′-AGGCGGAGTGTCTAAGTTCAA-3′ |
| PITX2-EX4F | 5′-CAGGAGATTTGGTGTGTCTGC-3′ |
| PITX2-EX4R | 5′-GGGAACTGTAATCTCGCAACC-3′ |
| PITX2-EX5F | 5′-AAAGCTGGCCCTGGTATCTT-3’ |
| PITX2-EX5R | 5′-CTCTCCCTTTCTTTAGTGCCC-3’ |
| **Primers for minigene assay** | |
| Oligo pCAS-KO1 -F | 5′-TGACGTCGCCGCCCATCAC-3′ |
| Oligo pCAS - 2R | 5′-ATTGGTTGTTGAGTTGGTTGTC-3′ |
| PITX2-PCAS-seq | 5′-GGCACCATGCTGGACGACATGC-3′ |
| PITX2-PCAS-seq2 | 5′-TCACACGGGCCGGTCCACTG-3′ |
| **Primers for qPCR** | |
| qGAPDH-F | 5′-CAAGGTCATCCATGACAACTTTG-3′ |
| qGAPDH-R | 5′-GTCCACCACCCTGTTGCTGTAG-3′ |
| qPITX2-F | 5′-GACCAACTGCCGCAAACTGG-3′ |
| qPITX2-R | 5′-GCCGCTTCTTCTTAGACGGG-3′ |

**Supplementary Table S2. *PITX2* variants identified in four Chinese families with ARS**

| Transcript | Case No. | Nucleotide change | Protein change | Type | AF of global  population  in gnomAD/ExAC | Novel variant reported |
| --- | --- | --- | --- | --- | --- | --- |
| NM_00120  4399.1 | Case 1 | c.118delA | p.Arg40Gly  fs*115 | frameshift | 0 | Yes |
|  | Case 2 | c.211G>T | p.Glu71X | nonsense | 0 | No[3] |
|  | Case 3 | c.253-1G>T | / | Splicing site | 0 | Yes |
|  | Case 4 | c.663_670dup  GACTCCTC | p.Pro224Arg  fs*18 | frameshift | 0 | Yes |

**References**

1. Soukarieh O, Gaildrat P, Hamieh M, Drouet A, Baert-Desurmont S, Frébourg T, Tosi M*, et al.* Exonic Splicing Mutations Are More Prevalent than Currently Estimated and Can Be Predicted by Using In Silico Tools. PLoS Genet 2016, 12: e1005756
2. Abramson J, Adler J, Dunger J, Evans R, Green T, Pritzel A, Ronneberger O*, et al.* Accurate structure prediction of biomolecular interactions with AlphaFold 3. Nature 2024, 630: 493-500
3. Wang X, Liu X, Huang L, Fang S, Jia X, Xiao X, Li S*, et al.* Mutation Survey of Candidate Genes and Genotype–Phenotype Analysis in 20 Southeastern Chinese Patients with Axenfeld–Rieger Syndrome. Current Eye Research 2018, 43: 1334-1341
